# The effectiveness of internal control and innovation performance: An intermediary effect based on corporate social responsibility

**DOI:** 10.1371/journal.pone.0234506

**Published:** 2020-06-11

**Authors:** Xiao Li

**Affiliations:** 1 Economics and Management School, Zhongyuan University of Technology, Zhengzhou, Henan, China; 2 Economics and Management School, Wuhan University, Wuhan, Hubei, China; The Bucharest University of Economic Studies, ROMANIA

## Abstract

From the perspective of the effectiveness of internal control, this study analyzes the influence of internal control on innovation performance and internal control on corporate social responsibility (CSR), and then analyzes the intermediary effect of CSR between internal control and innovation performance. The results show that the improvement of the effectiveness of internal control has a significant promoting effect on innovation performance, and promotes enterprises to strengthen CSR performance. Meanwhile, CSR activities take a significant intermediary effect in the process of improving innovation performance through internal control. Finally, it is suggested that state-owned enterprises and non-state-owned enterprises should communicate and cooperate, strengthen the construction of internal control system, and improve innovation performance and CSR practices. Furthermore, the intermediary effect of CSR activities in the process of improving innovation performance through internal control should be brought into play, so as to return the expectations and demands of stakeholders.

## 1. Introduction

Internal control and technological innovation are of great significance to the survival and development of enterprises. And the internal control has become an important system for Chinese enterprises to improve their social responsibilities [1]. Moreover, enterprises with strategic CSR achieve growth through both their product and their process innovations [2]. Technological innovation is the strategic support for building a modern economic system. Therefore, from the perspective of the effectiveness of internal control, it is of practical significance to explore the joint mechanism of internal control and CSR on innovation performance. From 2008 to 2010, Chinese five ministries and commissions, including the Ministry of Finance, successively issued the “Basic Norms for Enterprise Internal Control” and the “Supporting Guidelines for Enterprise Internal Control”, promoting the development of the construction of internal control. Where, the “Supporting Guidelines for Enterprise Internal control” include the “Application Guidelines for Internal Control”, the “Evaluation Guidelines for Internal Control” and the “Audit Guidelines for Internal Control”. These normative documents clearly point out that internal control is a process carried out by the board of directors, board of supervisors, managers and all employees to achieve the control objectives. The elements of internal control include the internal environment, risk assessment, control activities, information and communication, and internal supervision. As an institutional arrangement, internal control aims at reasonably ensuring the legal operation and compliance, safety of assets, and reliability of financial information, improving the efficiency and effect of operations, and thus promoting the realization of the development strategy of enterprises. Since the promulgation of the current standard system of internal control, Chinese listed enterprises have achieved some outstanding results in the implementation, evaluation and information disclosure of internal control, providing a good reference for the system construction of unlisted enterprises, and building a good micro foundation for the steady operation of the market economy. The improvement of the effectiveness of internal control can enhance the sustainable development of enterprises to a certain extent [3]. The effective internal control is conducive to improving the financing efficiency [4], and significantly and positively affects the investment efficiency of enterprises [5]. Moreover, the Chinese version of SOX system has a significant promoting effect on the improvement of financial performance of listed companies [6]. However, the internal control of some enterprises has not been really implemented. For instance, the auditors issued the negative audit reports of internal control on “Guitang Shares” and other enterprises [7]. Therefore, enterprises should still attach great importance to strengthening the construction of internal control standard system [8].

In the “Application Guidelines for Internal Control No. 10—Research and Development”, it is proposed that enterprises should attach importance to the research and development, combine their own development strategy, according to the requirements of market development and technological progress, formulate their scientific plans of research and development. The economic growth theory believes that the technological progress is an important driving force for the economic growth, and the key to the technological progress lies in the scientific and technological innovation. Enterprises are the main body of scientific and technological innovation, and the search for the factors driving innovation is the key to improving the driving force of economic growth [9]. As the output of innovation investment is characterized by uncertainty and untimeliness [10, 11], the sound internal control is needed to prevent those possible unclear R&D positioning, lack of capital management, and other moral hazard and adverse selection. In the current stage of Chinese economic development, enterprises need to strengthen the construction of internal control, to promote the steady development of capital market and national economy. Then, under the current background of internal control construction, how does internal control affect the status of technological innovation?

Meanwhile, the non-exclusivity of knowledge should be reasonably utilized to balance the private benefits and social benefits of innovators in the process of continuous operation [12]. The “Application Guidelines for Internal Control No. 4—Social Responsibility” has embedded CSR into the system of internal control. This guideline clearly states that enterprises should fulfill their social obligations and responsibilities in the process of operation and development, including production safety, product quality, environmental protection, resource conservation, promotion of employment, and protection of employees’ rights and interests. CSR refers to the responsibilities of enterprises to the creditors, government, customers, employees, community and other stakeholders as well as the environment according to a set of institutional arrangements while assuming economic responsibilities to shareholders [13]. Then, at the present stage, while promoting enterprises to realize the long-term development strategy, what impact does the construction of internal control have on CSR performance? Moreover, from the stakeholder theory, enterprises should actively pay attention to the expectations and appeals of different stakeholders. CSR activities play an important role in the sustainable development [14]. So, in the context of Chinese economic development entering a new normal, do CSR activities significantly promote technological innovation to further improve their own sustainable development?

**Fig 1:**
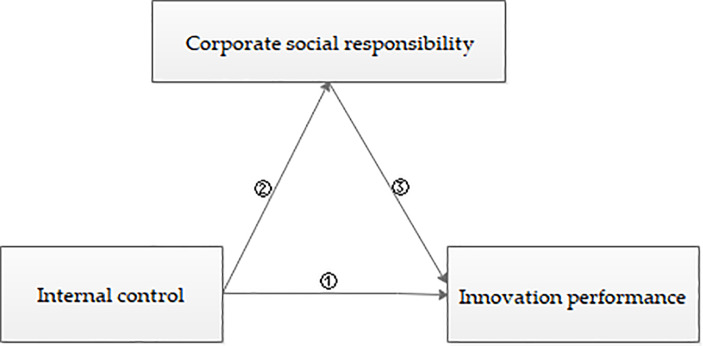
The research train of thought.

Based on the realistic consideration of the relationships between internal control and technological innovation, internal control and CSR performance, and CSR activities and technological innovation, from the perspective of the effectiveness of internal control, this study analyzes the influence of internal control on enterprise innovation performance and CSR performance. On this basis, this study explores the possible intermediary effect of CSR activities in the process of internal control influencing innovation performance, so as to strengthen the construction of internal control and CSR practices, and provide some empirical evidence and valuable suggestions for improving innovation performance under the background of current economic transformation. Fig 1 presents the research train of thought. Where, the symbol “①” represents the effect of internal control on innovation performance. “②” refers to the influence of internal control on CSR. And “③” indicates the possible intermediary effect of CSR activities between internal control and innovation performance. The remainder of this paper is organized as follows. Section 2 presents the literature review, theoretical basis, and research hypothesis. Section 3 presents the data source, variable definition, and model setting. Section 4 discusses the descriptive statistics and correlation of variables. Section 5 presents the model regression analysis. Section 6 develops the further analysis. Section 7 develops the robustness test. And section 8 concludes the study.

The possible contribution of this study is as follows. In the past, scholars tend to study the relationship between internal control and innovation performance. Some scholars have shown that internal control effectively prevents various risks in the process of technological innovation through the reasonable risk assessment, control and prevention [15, 16]. Besides, the existing literature tends to study the relationship between internal control and CSR performance. For instance, Ntim and Soobaroyen (2013) [17], Li et al. (2019) [18] believe that the partial content of internal control, as a specific system of corporate governance, still has a positive effect on CSR performance. However, there are few studies on the integration of internal control, CSR activities and innovation performance. On basis of the existing literature on the relationship between corporate governance and innovation performance [19, 20], this study introduces an intermediary variable–CSR performance, and explores deeply the intermediary effect of CSR activities between internal control and innovation performance. This study expands the mechanism of internal control, CSR activities and innovation performance, and enriches the existing literature on the relationship between internal control and innovation performance.

The possible practical significance of this study is mainly focused on the following aspects. (1) Internal control is an important factor influencing the innovation performance and CSR activities. To strengthen the innovation performance and CSR activities, enterprises should consider enhancing the effectiveness of internal control. (2) The effect of internal control on innovation performance is subject to different property rights. In non-state-owned enterprises, internal control plays a positive role in promoting innovation performance. However, in state-owned enterprises, internal control has not yet shown a significant promoting effect on innovation performance. (3) CSR activities take an intermediary effect between internal control and innovation performance, but this intermediary effect is only reflected in non-state-owned enterprises.

## 2. Literature review, theoretical basis, and research hypothesis

### 2.1 Internal control and innovation performance

The essence of innovation is the innovation of human knowledge, science and technology. However, under the condition of information asymmetry, the insiders’ opportunistic behaviors lead to the deviation of innovation investment from maximizing shareholders’ interests, leading to the low innovation efficiency of enterprises [21]. The promotion of enterprises’ innovation ability depends on the effective input mechanism, and the integration of human capital and material capital into the innovation activities is often determined by the internal system of enterprises [19]. The influence of internal control on innovation performance can be divided into two viewpoints, namely the promotion theory of internal control and the paradox of internal control. The promotion theory of internal control holds that the strengthening of internal control contributes to the improvement of technical innovation output [22, 23]. The paradox of internal control holds that the excessive institutionalization often leads to the rigid management, which is not conducive to the implementation of flexible technological innovation [24, 25]. According to Liu et al. (2018) [26], the internal risk management and control ability is significantly and negatively correlated with innovation efficiency, indicating that the paradox of internal control may exist in innovation-oriented enterprises. And on the whole, internal control has a promoting effect on innovation input and innovation performance, but the promoting force is weak [27]. But internal control is implemented late in China, and the overly strict system is not established. The promotion theory of internal control may be more suitable for Chinese national conditions [28]. Also, Chen et al. (2018) [20], Yang et al. (2019) [29], Li and Shi (2019) [30] affirm the appropriateness of the promotion theory of internal control.

The behavior control has a positive moderating effect on the relationship between experimental learning and indigenous innovation [31]. Chinese “Application Guidelines for Internal Control No. 10—Research and Development” aims to promote enterprises to achieve the independent innovation, enhance their core competitiveness, effectively control the risk of R&D activities, and promote the strategy of long-term development. Internal control can effectively reduce the uncertainty of technological innovation by evaluating, controlling and preventing risks [15]. Shi et al. (2014) [32] believe that on the basis of improving the operation mechanism of internal control, enterprises should take the continuous innovation as the long-term development objective, stimulate employees’ sense of innovation, and then actively identify, digest, absorb and integrate the cutting-edge knowledge. Enterprises should attach importance to R&D activities, scientifically formulate R&D plans according to the requirements of market development and technological progress, and strengthen the management of the whole process of R&D activities. On basis of standardizing R&D behaviors, the transformation and effective utilization of R&D results should be promoted to continuously improve the independent innovation abilities of enterprises. Internal control is an internal management system, path and intermediate mechanism to achieve the governance objectives, which can effectively alleviate the agency problem and information asymmetry at the operation level of R&D investment [21, 33]. In the “Application Guidelines for Internal Control No. 10—Research and Development”, the approval of R&D project, R&D personnel allocation, management of R&D process, and transformation of R&D results are specified in detail. Through the strict monitoring and intervention, budget and assessment mechanism, the unreasonable R&D projects in the innovation activities can be restrained, and the strategic positioning and risk tolerance boundary of an organization can be clearly conveyed to the employees. Furthermore, it builds a good internal environment for innovation activities and stimulates innovation at all levels within the organization. Therefore, as an important influencing factor of technological innovation, the effective internal control shall play a positive role in promoting innovation performance.

Based on the above analysis, the following research hypothesis is proposed.

**Hypothesis 1.** The improvement of the effectiveness of internal control will significantly improve the innovation performance of enterprises.

### 2.2 Internal control and CSR performance

Enterprises should combine their own interests with employees’ health and social progress while pursuing entrepreneurs’ profits. The goal of corporate governance has experienced a process from the maximization of profit to the maximization of shareholder equity, and then to the maximization of stakeholder benefit. The stakeholder theory emphasizes that while promoting their own profits, enterprises should take into account the corresponding responsibilities for employees, consumers, communities and social development, which achieves a reasonable integration of corporate interests and social effects [34, 35]. CSR is a strategic move that helps to change stakeholders’ perceptions of enterprises and expectations of performance, deliver signals of performance improvement, and divert society’s attention to enterprises [36]. Barnett and Salomon (2012) [37] found that the relationship between CSR performance and enterprises’ financial performance is U-shaped, enterprises with low CSR performance have higher financial performance than enterprises with moderate CSR performance, but enterprises with high CSR performance have the highest financial performance. CSR input can convert to higher benefits only when it generates a solid relationship between enterprises and their stakeholders [38]. When the power supervision between the governance and the management is put in place to achieve higher internal governance, it is conducive to improving CSR performance. Internal control promotes the optimization of capital allocation efficiency, thus promoting the realization of sustainable growth. However, Qin (2019) [39] believes that there is no significant relationship between internal control and CSR activities. But the risk of social responsibility arising from stakeholders must not be ignored. The uncontrolled risk of social responsibility will have a serious adverse impact on the sustainable growth of enterprises [40]. The risk derived from CSR aggravates the risk level of enterprises and broadens the risk boundary faced by enterprises.

Hao et al. (2018) [1] believe that internal control has a significant and partial moderating effect between CSR and stock price crash risk. For enterprises, it is necessary to effectively control the significant behavioral risks that deviate from their established objectives [41]. The significance of CSR activities lies in the transcendence of the governance model of shareholder supremacy. Enterprises should build a governance mechanism to actively carry out CSR activities on basis of reshaping the governance structure to strengthen their internal system. The effective internal control not only alleviates the conflict of interest caused by those uncoordinated governance structure, but also reasonably guarantees the efficiency of business activities, the reliability of financial reports and the compliance with laws and regulations. The “Application Guidelines for Internal Control No. 4—Social Responsibility” clearly states that enterprises should strengthen the construction of internal control in production safety, product quality, environmental protection, resource conservation, employment promotion and employee interests. This implies that CSR performance has been integrated into the construction process of internal control. Thereby, the effective internal control can help make up for the lack of CSR activities, and exert a positive effect on improving the disclosure quality of CSR information [42, 43]. The effective internal control alleviates CSR risks, protects the legitimate rights and interests of stakeholders, and promotes the successful realization of the strategic objectives of CSR practices. Generally, the internal control can be taken as an institutional system of risk management. When the operation of internal control is effective, it can prevent the improper behaviors that damage the reputation and image, and avoid the negative events that damage CSR practices, to improve the realistic performance of CSR activities. Internal control can promote enterprises to fulfill their social responsibilities by reducing agency costs [18]. The objectives of internal control are in line with the expectations and demands of stakeholders. Therefore, the effective internal control can enhance the practical value of CSR activities. Furthermore, on basis of ensuring the effective implementation of internal control, enterprises will actively carry out CSR activities and improve their CSR performance.

Based on the above analysis, the following research hypothesis is proposed.

**Hypothesis 2.** The enhancement of the effectiveness of internal control is conducive to the significant improvement of CSR performance.

### 2.3 Internal control, CSR and innovation performance

As an endogenous monitoring mechanism, the effective internal control alleviates the problem of information asymmetry between enterprises and stakeholders by fully linking enterprises and markets [44–46]. Also, the effective internal control strengthens the efficiency and quality of information communication, forms a good system of division of responsibilities and checks and balances of power, alleviates the conflict between enterprises and stakeholders, and improves the protection of the rights and interests of stakeholders, thus promoting enterprises to actively improve CSR performance [17, 18]. CSR activities mean to satisfy the value demands of stakeholders, thus maintaining the relationship between enterprises and stakeholders. The social responsibility behavior based on stakeholder orientation is conducive to establishing and maintaining a good relationship between enterprises and stakeholders, and supports enterprises to obtain the necessary operational resources from stakeholders [47, 48]. Meanwhile, the production of products with CSR attribute enables enterprises to realize a differentiation strategy, and CSR activities become a resource with competitive advantages [49]. The improvement of CSR performance helps to obtain the information and resources needed in the process of technological innovation [50, 51]. Wang et al. (2019) [52] believe that there is a positive association between CSR and innovation performance in high-polluting firms. Under the circumstance that enterprises choose CSR as a competitive tool, CSR has a significant and positive effect on innovation performance [53]. However, Li et al. (2018) [51] believe that CSR activities have an inverted U-shaped influence on enterprise technological innovation, which can effectively promote enterprise innovation within a certain critical point, but hinder enterprise innovation beyond that critical point.

The activities of technological innovation run through the whole process of R&D decision-making, R&D input, product testing and final innovative products [54]. In the process of technological innovation, the design, testing and use of innovation links are inseparable from the support of stakeholders. Enterprises can obtain more diversified sources of innovative knowledge by assuming CSR to diversified stakeholders [55]. Moreover, through CSR practices, enterprises establish a direct relationship with external stakeholders such as the government, suppliers and customers, thus expanding the sources of external information and knowledge [56]. Enterprises’ acquisition and utilization of knowledge promote the development of technological innovation ability. The explicit or implicit knowledge acquired by enterprises from stakeholders plays a key role in the construction of innovation ability [57]. For instance, customers take a more direct role in the reverse catalysis of technological innovation, which can provide the source power for enterprise innovation and play a key role in the early market stage of new products [58]. The external stakeholders often have some new, non-redundant professional knowledge and skills, which can be used to supplement the internal knowledge of enterprises, thus helping to improve the technological innovation of enterprises [59, 60]. Therefore, CSR activities promote a good relationship between enterprises and external stakeholders, and then help enterprises acquire the knowledge and technology owned and accessible by these stakeholders, so as to improve the performance of technological innovation.

Meanwhile, both attitudes and need statements of jobs are affected by informational social influence [61]. Through the active CSR practices, enterprises can convey superior values to the market of technical personnel, which shows that they pay great attention to and attach importance to the interests of stakeholders. In this case, it can make job-seekers feel the expected superior working atmosphere, promote enterprises to introduce more high-quality technical R&D personnel, and reserve human capital for improving the innovation performance. Stakeholders derive functional, psychological and value satisfaction benefits from CSR activities [62]. From the “people-oriented” concept, as the stakeholders of enterprises, R&D personnel are treated fairly and with high quality, and become the direct beneficiaries of CSR activities. In the process of CSR activities, R&D personnel acquire practical skills and experience, and apply relevant skills and experience to their work to obtain the functional benefits. When CSR activities involve the life field concerned by R&D personnel, they connect the work and life through CSR activities to achieve a benign transformation between the work environment and living environment, relieve pressure and discomfort at work, and gain the psychological benefits. Moreover, CSR activities convey shared values to R&D personnel, enabling them to express themselves more freely in their work [63], so as to obtain the value satisfaction benefits. Therefore, enterprises integrate the expectations of R&D personnel into CSR strategy, enhance their recognition and loyalty to the organization, motivate their work enthusiasm and innovation ability, so as to select high-quality innovation projects, formulate scientific innovation plans, and ultimately improve the efficiency of transforming R&D input into innovation results. When enterprises strategically undertake social responsibility, they achieve growth through product and process innovation [2]. Furthermore, considering the hypothesis that the improvement of the effectiveness of internal control significantly strengthens CSR performance (hypothesis 2 above), this study forms an intermediate action path for internal control to affect enterprise innovation performance, that is, CSR activities take an intermediary effect in the process of internal control to improve the innovation performance.

Based on the above analysis, the following research hypothesis is proposed.

**Hypothesis 3.** CSR activities take an intermediary effect significantly between the effective internal control and enterprise innovation performance.

## 3. Data source, variable definition and model setting

### 3.1 Data source

No doubt that the intensity of R&D investment affects enterprise innovation performance. The disclosed data on the intensity of R&D investment (i.e. the proportion of R&D investment in operating revenue) are more concentrated in the period after 2011. In view of the availability of data, those companies listed on the Shanghai and Shenzhen stock markets are selected as the research sample during 2012–2017. The data are obtained from China stock exchange database, Wind database, and DIB internal control and risk management database. The sample data shall be excluded according to the following criteria. (1) Financial and insurance enterprises. (2) ST, *ST class. (3) Lack of indicator data. On this basis, the continuous variables are Winsorized with two‐way 1% quantiles, to avoid the adverse effect of abnormal observations on the analyses.

### 3.2 Variable definition

#### 1. Interpreted variable

(1) For the evaluation of innovation performance, the number of patent applications can reflect the true innovation ability of enterprises [64]. And the data of patent application is reliable [65]. With reference to the research of Guan and Gao (2009) [66], Berchicci (2013) [67], Maggitti et al. (2013) [68], Tian and Wang (2014) [69], the number of patent applications of enterprises is adopted to measure the innovation performance. That is the sum of the number of inventions, utility models and appearance designs in a year. And the variable symbol is denoted as “PATENT”. The more the number of patent applications annually, the higher the performance of innovation output.

(2) For the measure of CSR performance, Shanghai stock exchange in China issued the “Notice on Strengthening the Social Responsibility of Listed Enterprises” in 2008, aiming to guide listed enterprises to actively fulfill their social responsibilities, attach importance to the common interests of stakeholders, and contribute to the construction of harmonious society and the sustainable development of social economy. The notice puts forward the indicator of “social contribution value per share” (SCPS) for the first time, providing a new and important benchmark for the comprehensive and objective evaluation of enterprises’ value creation. The disclosure of SCPS information helps the public to have a more comprehensive understanding of the true value that enterprises create for their shareholders, employees, customers, creditors, communities and the society as a whole. Therefore, from the authority and universality of indicators, this study refers to the research design of Fan et al. (2014) [70] and Li et al. (2018) [71], and adopts SCPS as the measurement index of CSR performance. In this regard, SCPS is determined as formula (1).

SCPS=CS (1)

For formula (1), C = C_1_+C_2_+C_3_+C_4_+C_5_-C_6_+C_7_+C_8_-C_9_. The meanings of C_1_ ~ C_5_ are the net margin, income tax expense, business tariff and annex, cash payments to and for employees, employee compensation payable at the end of this period, respectively. C_6_ represents the employee compensation payable at the end of last term. The meanings of C_7_, C_8_ are the finance charges and donation outlay, respectively. And C_9_ represents the sewage charges and cleaning charges. In the numerator of formula (1), the data of “donation outlay” are manually collected from the “non-operating expenses” itemized in the notes to the financial statements. The data of “sewage charges and cleaning charges” are collected manually from the detailed item of “administrative expenses” in the notes to the financial statements. The denominator S of formula (1) represents the average of the total number of shares at the beginning and end of this period.

#### 2. Explanatory variable

Driven by control objectives, the effectiveness of internal control includes the effectiveness of design and the effectiveness of operation. It includes not only the effectiveness of preventing, finding and correcting the major misstatements in the financial reports, but also the effectiveness of restraining the executive behaviors and thereby reducing agency costs [72]. Since the release of the DIB internal control index in 2011, it has been widely recognized by the academia, practice and regulatory departments. Based on the status quo of internal control implemented by listed enterprises, the basic index of internal control is designed in accordance with the realization degree of the five control objectives of compliance, report, asset safety, operation and strategy. Furthermore, internal control defects are used as the correction variables to modify the basic index, and then DIB internal control index is formed that comprehensively reflects the level of internal control and ability of risk control of listed enterprises. Therefore, for the explanatory variable in the models—the effectiveness of internal control (IC), the internal control index of DIB listed enterprises is adopted as the indicator to evaluate the effectiveness of internal control of enterprises. The greater is the index, the more effective is the implementation of internal control.

#### 3. Control variable

In terms of the selection of control variables, given that R&D investment intensity reflects the amount of resources invested in innovation business, and at home and abroad, there are three main ways to measure R&D investment intensity, which are the proportions of R&D investment in operating revenue, market value and total assets. In this study, the percentage of R&D investment in operating revenue is adopted to evaluate the R&D investment intensity, to investigate its influence on enterprise innovation performance. The reason for this adoption is that enterprises are more willing to carry out technological innovation only on basis of the income, and this approach is more in line with the principle of matching income and expenditure. Meanwhile, the degree of equity concentration will affect the principal-agent relationship among stakeholders and determine the distribution of control rights of listed enterprises. Therefore, the ownership concentration (SHRCR) is taken as a control variable to evaluate its possible impact on enterprise innovation performance and CSR practices. Besides, the higher growth rate of operating revenue implies the strong competitiveness of products to some extent. Through the profits obtained, enterprises can carry out the R&D activities of new products to continuously meet the needs of consumers in the market, which is conducive to the realization of sustainable development and has an impact on CSR activities. Thus, the influence of sales growth rate (GROWTH) is controlled. Meanwhile, the relevant study of Chen et al. (2018) [20] is referenced, the influences of return on total assets (ROA), equity ratio (LER), size of the board of supervisors (SUPERVISOR), double duty (DUAL), executive compensation (LNSALARY), comprehensive leverage (DTL), asset size (LnASSET), audit opinion (AUDIT) and property attribute (STATE) are controlled. The industry effect and annual effect are also controlled in the regression analyses. Table 1 presents the name and calculation method of each variable.

**Table 1:** Variable name and definition.

Nature	Symbol	Name	Calculation method
Explained variable	PATENT	Innovation performance	The sum of the applications of inventions, utility models and appearance designs in a year
	SCPS	CSR performance	Social contribution per share. The calculation method is shown in formula (1).
Explanatory variable	IC	Effectiveness of internal control	DIB ∙ internal control index of listed companies
Control variable	R&D	Intensity of R&D investment	The proportion of R&D investment in operating revenue
	ROA	Return on total assets	Net profit/average total assets
	LER	Equity ratio	Liabilities/owners’ equity
	GROWTH	Sales growth rate	(Current operating income—last operating income)/last operating income
	SHRCR	Ownership concentration	Shareholding ratio of the top 10 shareholders
	SUPERVISOR	Size of the board of supervisors	Total number of supervisors
	DUAL	Double duty	When the chairman concurrently serves as the general manager, the value is 1; otherwise 0
	LNSALARY	Executive compensation	Natural logarithm of total compensation of directors, supervisors and top three executives
	DTL	Comprehensive leverage	Financial leverage × operating leverage
	LnASSET	Asset size	Natural logarithm of total assets at the beginning of the year
	AUDIT	Audit opinion	Dummy variable set according to the type of audit opinion in the disclosed annual audit report. Where, the value of standard unreserved opinion is 1; otherwise 0
	STATE	Property attribute	Dummy variable, if state-owned enterprise, 1; otherwise 0
	YEAR	Year	The annual effect
	IND	Industry	Industry effect. According to the “Guidelines on Industry Classification of Listed Companies (2012 revision)” issued by China Securities Regulatory Commission, the sample enterprises are divided into 17 industries, and a total of 16 industry dummy variables are set
	ε		Random disturbance term

### 3.3 Model setting-up

In order to verify the rationality of the hypotheses mentioned above, the relevant studies of Hazarika et al. (2012) [72], Chen et al. (2018) [20], Li et al. (2018) [71] are referenced, the following models 1, 2 and 3 are constructed. The regression estimation of panel data for parameters is conducted, respectively, to test the above hypotheses 1, 2 and 3. Furthermore, in order to avoid the adverse impact of dimensional differences between the explained variable and explanatory variable on the results, in model 1—model 3, the value of the explanatory variable (IC) is taken as 1, when the DIB index of an enterprise in this period is higher than that of last period, indicating that the effectiveness of the enterprise’s internal control tends to improve. Otherwise, the value is 0, indicating that the effectiveness of internal control remains unchanged or decreases. Meanwhile, in model 1—model 3, the value of the explained variable (LnPATENT) is the natural logarithm of 1 plus the total number of annual patent applications. Besides, to alleviate the endogenous problem caused by the reverse causality, the control variables including R&D, ROA, LER, GROWTH, LNSALARY, DTL and AUDIT are taken as the first-order lag values.

**Model 1**.

$$\begin{array}{l} {\mathrm{LnPATENT}}_{i,t}=\alpha_{0}+\alpha_{1}{\mathrm{IC}}_{i,t}+\alpha_{2}R\&D_{i,t‐1}+\alpha_{3}{\mathrm{ROA}}_{i,t‐1}+\alpha_{4}{\mathrm{LER}}_{i,t‐1}+\alpha_{5}{\mathrm{GROWTH}}_{i,t‐1}+\alpha_{6}{\mathrm{SHRCR}}_{i,t}+\\ \;\alpha_{7}{\mathrm{SUPERVISOR}}_{i,t}+\alpha_{8}{\mathrm{DUAL}}_{i,t}+\alpha_{9}{\mathrm{LNSALARY}}_{i,t‐1}+\alpha_{10}{\mathrm{DTL}}_{i,t‐1}+\alpha_{11}{\mathrm{LnASSET}}_{i,t}+\\ \;\alpha_{12}{\mathrm{AUDIT}}_{i,t‐1}+\alpha_{13}{\mathrm{STATE}}_{i,t}+\alpha_{14}\sum_{t}\mathrm{YEAR}+\alpha_{15}\sum_{t}\mathrm{IND}+\varepsilon_{i,t} \end{array}$$ (2)

**Model 2**.

$$\begin{array}{l} {\mathrm{SCPS}}_{i,t}=\beta_{0}+\beta_{1}{\mathrm{IC}}_{i,t}+\beta_{2}{\mathrm{R\&D}}_{i,t‐1}+\beta_{3}{\mathrm{ROA}}_{i,t‐1}+\beta_{4}{\mathrm{LER}}_{i,t‐1}+\beta_{5}{\mathrm{GROWTH}}_{i,t‐1}+\beta_{6}{\mathrm{SHRCR}}_{i,t}+\\ \;\beta_{7}{\mathrm{SUPERVISOR}}_{i,t}+\beta_{8}{\mathrm{DUAL}}_{i,t}+\beta_{9}{\mathrm{LNSALARY}}_{i,t‐1}+\beta_{10}{\mathrm{DTL}}_{i,t‐1}+\beta_{11}{\mathrm{LnASSET}}_{i,t}+\\ \;\beta_{12}{\mathrm{AUDIT}}_{i,t‐1}+\beta_{13}{\mathrm{STATE}}_{i,t}+\beta_{14}\sum_{t}\mathrm{YEAR}+\beta_{15}\sum_{t}\mathrm{IND}+\varepsilon_{i,t} \end{array}$$ (3)

**Model 3**.

$$\begin{array}{l} {\mathrm{LnPATENT}}_{i,t}=\gamma_{0}+\gamma_{1}{\mathrm{IC}}_{i,t}+\gamma_{2}{\mathrm{SCPS}}_{i,t}+\gamma_{3}{\mathrm{R\&D}}_{i,t‐1}+\gamma_{4}{\mathrm{ROA}}_{i,t‐1}+\gamma_{5}{\mathrm{LER}}_{i,t‐1}+\gamma_{6}{\mathrm{GROWTH}}_{i,t‐1}+\\ \;\gamma_{7}{\mathrm{SHRCR}}_{i,t}+\gamma_{8}{\mathrm{SUPERVISOR}}_{i,t}+\gamma_{9}{\mathrm{DUAL}}_{i,t}+\gamma_{10}{\mathrm{LNSALARY}}_{i,t‐1}+\gamma_{11}{\mathrm{DTL}}_{i,t‐1}+\\ \;\gamma_{12}{\mathrm{LnASSET}}_{i,t}+\gamma_{13}{\mathrm{AUDIT}}_{i,t‐1}+\gamma_{14}{\mathrm{STATE}}_{i,t}+\gamma_{15}\sum_{t}\mathrm{YEAR}+\gamma_{16}\sum_{t}\mathrm{IND}+\varepsilon_{i,t} \end{array}$$ (4)

**Fig 2:**
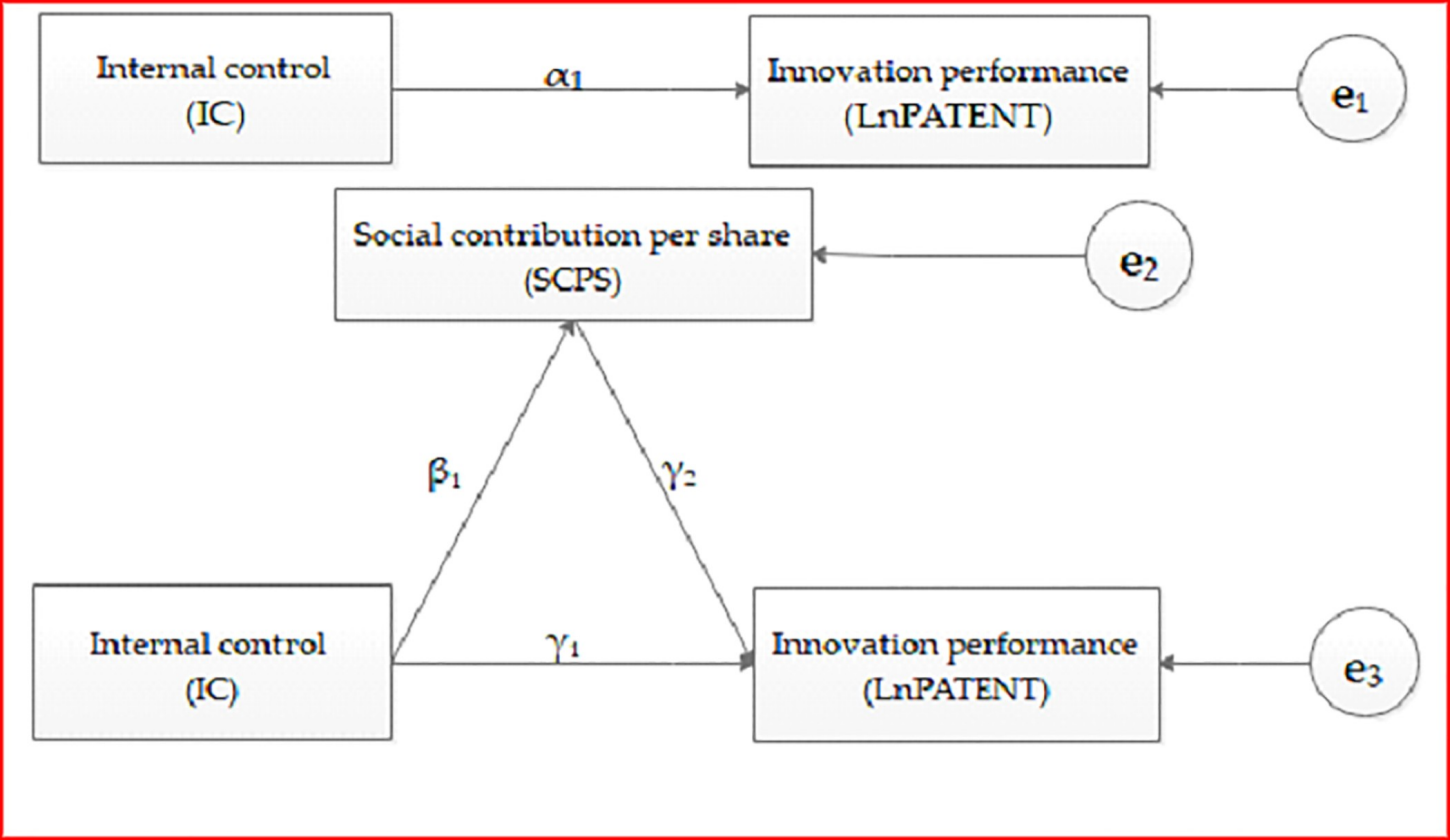
The diagram of intermediary effect.

Model 1—model 3 are also used to examine the possible intermediary effect of CSR activities in the process of internal control promoting innovation performance, which can be described by the diagram of intermediary effect (Fig 2). Where, the coefficient α_1_ in model 1 is the total effect of internal control (IC) on innovation performance (LnPATENT). The coefficient β_1_ in model 2 refers to the effect of internal control (IC) on the intermediary variable—Social contribution per share (SCPS). The coefficient γ_2_ in model 3 refers to the effect of the intermediary variable (SCPS) on the explained variable—innovation performance (LnPATENT) after the effect of internal control (IC) is controlled. And in model 3, the coefficient γ_1_ is the direct effect of internal control (IC) on the explained variable (LnPATENT) after the effect of the intermediary variable (SCPS) is controlled.

## 4. Descriptive statistics and correlation of variables

### 4.1 Descriptive statistics

Table 2 reports the descriptive statistical results of variables. For the explained variable in model 1 and model 3, the standard deviation of PATENT is 167.710, and the minimum (maximum) of PATENT is 1 (1138), indicating that the number of annual patent applications of different sample enterprises is quite different. After the number of patent applications is taken into the natural logarithm, the standard deviation of LnPATENT is 1.381, and the mean (median) of LnPATENT is 3.533 (3.555). The variation amplitude of this variable tends to decrease, which can avoid the adverse effect on the following regression analyses. For the explained variable in model 2, the standard deviation of SCPS is 1.154, and the mean (median) of SCPS is 1.379 (1.054), indicating that the sample enterprises have assumed their due social responsibilities to different degrees, and CSR performance of the sample enterprises has a great variability. However, the minimum of SCPS is -0.209, implying that the existence of negative CSR performance in sample enterprises, and there may be some negative response to CSR activities, which will have a negative impact on the overall reputation of enterprises.

**Table 2:** The descriptive statistics table of variables.

Variable	Mean	Median	Maximum	Minimum	Deviation	Observations
PATENT	88.154	35.000	1138.000	1.000	167.710	4308
LnPATENT	3.533	3.555	7.037	0.000	1.381	4308
SCPS	1.379	1.054	6.230	-0.209	1.154	4304
IC	649.341	673.015	863.140	0.000	128.960	4308
R&D	4.424	3.515	26.900	0.070	4.259	4308
ROA	0.044	0.036	0.214	-0.116	0.051	4308
LER	1.080	0.767	6.330	0.066	1.059	4308
GROWTH	0.154	0.107	2.042	-0.376	0.326	4308
SHRCR	55.208	55.132	87.412	23.472	14.306	4308
SUPVERSIOR	3.705	3.000	7.000	3.000	1.124	4308
DUAL	0.244	0.000	1.000	0.000	0.429	4308
LNSALARY	14.451	14.411	16.193	13.009	0.639	4308
DTL	2.514	1.531	23.498	-1.984	3.401	4308
LnASSET	22.247	22.050	26.054	20.223	1.198	4308
AUDIT	0.984	1.000	1.000	0.000	0.126	4308
STATE	0.426	0.000	1.000	0.000	0.495	4308

For the explanatory variable in model 1—model 3, the mean (median) of IC is 649.341 (673.015), indicating that after the promulgation and implementation of the “Supporting Guidelines for Enterprise Internal Control”, the implementation of internal control and the standardization and integrity of information disclosure are generally good, and most enterprises have realized the importance of effective operation of internal control. However, the standard deviation of IC is 128.960, and the maximum (minimum) of IC is 863.140 (0.000). The effectiveness of internal control in the sample enterprises is uneven. During the observation period, there are some cases of internal control failure in some enterprises, and the effectiveness of internal control of these enterprises needs to be further improved.

For the control variables, the mean (standard deviation) of R&D is 4.42% (4.259), and the maximum (minimum) of R&D is 26.90% (0.07%), indicating that the proportion of R&D input in the operating revenue varies to a certain extent, and the emphasis of different sample enterprises on R&D business is obviously different. The minimum of ROA is -0.116, and the standard deviation of LER (GROWTH) is 1.059 (0.326). The above results show that the operating income, liabilities and growth of listed enterprises in China are different. The median of SHRCR is 55.13%, and the average shareholding ratio of top 10 shareholders in the sample enterprises is 55.21%. The mean of DUAL is 24.37%, and the board of supervisors shall be composed of at least 3 supervisors and at most 7 supervisors. The standard deviations of LNSALARY, DTL and LnASSET are 0.639, 3.401 and 1.198, respectively. In addition, 42.60% of the sample enterprises are state-owned. The mean of AUDIT is 0.984. During the observation period, the external auditors take a positive attitude towards the legality and fairness of financial reports of nearly 98.40% of the sample enterprises, which ensures the reliability of the data used in this study.

### 4.2 Correlation of variables

Table 3 presents the correlation coefficients of variables. For model 1 and model 3, the correlation coefficient between the explanatory variable (IC) and the explained variable (LnPATENT) is 0.102 (*p <* 0.01). This result, to some extent, supports hypothesis 1 above, indicating that the effective internal control is conducive to improving enterprises’ innovation performance. For model 2, the correlation coefficient between the explanatory variable (IC) and the explained variable (SCPS) is 0.243 (*p <* 0.01), implying that internal control can be a driving force for CSR practices. Hypothesis 2 above is initially supported. For model 3, the correlation coefficient between SCPS and LnPATENT is 0.277 (*p <* 0.01), suggesting that the strengthening of CSR practices can be a favorable factor to enhance enterprises’ innovation performance. Meanwhile, with the elaboration of hypothesis 2, CSR activities are expected to present an intermediary effect significantly between internal control and innovation performance. Hypothesis 3 above is preliminarily supported.

**Table 3:** The table of correlation coefficients among variables.

Variable	LnPATENT	SCPS	IC	R&D	ROA	LER	GROWTH	SHRCR	SUPVERSIOR	DUAL	LNSALARY	DTL	LnASSET	AUDIT
LnPATENT	1.000													
SCPS	0.277***	1.000												
IC	0.102***	0.243***	1.000											
R&D	0.049***	-0.106***	-0.014	1.000										
ROA	0.062***	0.461***	0.341***	0.050***	1.000									
LER	0.142***	0.141***	-0.105***	-0.266***	-0.353***	1.000								
GROWTH	0.052***	0.158***	0.113***	-0.001	0.242***	-0.016	1.000							
SHRCR	0.094***	0.242***	0.117***	-0.113***	0.155***	-0.005	0.090***	1.000						
SUPVERSIOR	0.140***	0.189***	0.004	-0.117***	-0.025	0.191***	-0.054***	0.060***	1.000					
DUAL	-0.049***	-0.078***	-0.001	0.118***	0.049***	-0.139***	0.011	-0.045***	-0.149***	1.000				
LNSALARY	0.284***	0.349***	0.150***	0.047***	0.310***	-0.036**	0.065***	0.055***	0.056***	-0.008	1.000			
DTL	-0.018	-0.100**	-0.030*	-0.091**	-0.222**	0.212**	-0.083**	-0.051***	0.062***	-0.043***	-0.126***	1.000		
LnASSET	0.443***	0.362***	0.090***	-0.282***	-0.052***	0.437***	-0.087***	0.199***	0.320***	-0.170***	0.366***	0.137***	1.000	
AUDIT	0.055***	0.071***	0.389***	-0.024	0.135***	-0.063***	0.048***	0.036**	-0.006	0.016	0.025	-0.015	-0.014	1.000
STATE	0.113***	0.213***	-0.019	-0.170***	-0.150***	0.296***	-0.093***	0.065***	0.376***	-0.288***	-0.001	0.101***	0.357***	0.002

*** Significant at 1%

** Significant at 5%

* Significant at 10%.

Among the control variables in model 1 and model 3, ROA (0.062), LER (0.142), GROWTH (0.052), SHRCR (0.094), SUPERVISOR (0.140), LNSALARY (0.284), LnASSET (0.443), AUDIT (0.055), STATE (0.113) are positively and significantly (*p* < 0.01) correlated with the explained variable (LnPATENT), respectively. DUAL (-0.049) is negatively and significantly (*p* < 0.01) correlated with the explained variable (LnPATENT). These reported results of correlation coefficients ensure the rationality of model 1 and model 3 above. Also, among the control variables in model 2, ROA (0.461), LER (0.141), GROWTH (0.158), SHRCR (0.242), SUPERVISOR (0.189), LNSALARY (0.349), LnASSET (0.362), AUDIT (0.071), STATE (0.213) are positively and significantly (*p* < 0.01) correlated with the explained variable (SCPS), respectively. R&D (-0.106, p < 0.01), DUAL (-0.078, p < 0.01), DTL (-0.100, p < 0.05) are negatively and significantly (*p* < 0.01) correlated with the explained variable (SCPS), respectively. These reported results of correlation coefficients ensure the rationality of model 2 above.

Also, it is shown in Table 3 that the maximum absolute value of the correlation coefficients is 0.437. This maximum exists between LnASSET and LER, which is less than the threshold of 0.800. Taken together, it is shown that there is no severe multicollinearity in the model 1—model 3, which provides a reliable guarantee for the regression analyses below.

## 5. Model regression analysis

The descriptive statistics for single variable and correlation coefficients between variables are presented above. But they are only preliminary analyses because the other factors that affect the explained variables are not included. The data type used in this study is panel data. Panel data analysis has a certain advantage for alleviating the endogenous problem caused by missing variables that do not vary with time. The specific regression analysis methods include the mixed OLS method, fixed effect model and random effect model. LSDV method is adopted to analyze model 1—model 3, and the null hypothesis that “the coefficients of all individual dummy variables are zero” is rejected, indicating the existence of individual fixed effect. The robust Hausman tests for model 1—model 3 show that the Sargan-Hansen *χ*^2^ are 315.722 (*p = 0*.*000*), 415.706 (*p = 0*.*000*), 329.610 (*p = 0*.*000*), respectively, indicating that the fixed effect model should be adopted instead of random effect. Accordingly, the regression estimation results of model 1—model 3 are shown in Table 4.

**Table 4:** Statistical results of regression coefficients for model 1—model 3.

Variable	Model 1	Model 2	Model 3
	Coefficient (S.E.)	Coefficient (S.E.)	Coefficient (S.E.)
Intercept	-4.791** (1.873)	3.325* (1.819)	-5.289*** (1.841)
IC	0.046** (0.023)	0.064*** (0.017)	0.040* (0.023)
SCPS			0.135*** (0.036)
L.R&D	0.023* (0.013)	0.002 (0.008)	0.019 (0.016)
L.ROA	2.103*** (0.526)	1.358*** (0.364)	1.892*** (0.532)
L.LER	-0.004 (0.055)	0.214*** (0.044)	-0.032 (0.055)
L.GROWTH	-0.028 (0.050)	0.201*** (0.039)	-0.060 (0.051)
SHRCR	0.003 (0.003)	0.015*** (0.003)	0.001 (0.003)
SUPERVISOR	-0.055 (0.047)	-0.050 (0.043)	-0.052 (0.047)
DUAL	0.224*** (0.056)	0.016 (0.042)	0.218*** (0.056)
L.LNSALARY	0.044 (0.065)	0.064 (0.048)	0.035 (0.065)
L.DTL	-0.001 (0.004)	-0.011*** (0.003)	-0.0001 (0.004)
LnASSET	0.319*** (0.070)	-0.173** (0.067)	0.346*** (0.068)
L.AUDIT	-0.007 (0.174)	-0.024 (0.165)	-0.013 (0.169)
STATE	-0.135 (0.209)	0.022 (0.128)	-0.140 (0.207)
YEAR/ IND	YES	YES	YES
# of obs.	3590	3586	3586
Within_R^2^	0.117	0.159	0.125
F_Value	15.69***	22.34***	16.18***

*** Significant at 1%

** Significant at 5%

* Significant at 10%.

Robust standard errors in brackets are clustered at the enterprise level.

### 5.1 Analyses of model 1’s regression results

#### 5.1.1 Analyses of model 1’s explanatory variable

As shown in column 2 of Table 4, in model 1, the coefficient on the explanatory variable (IC) is positive and significant (0.046, *p* < 0.05). This result shows that the more effective the implementation of internal control is, the higher the innovation output of the enterprise is. The improvement of the effectiveness of internal control has a significant and positive impact on the innovation performance. Hypothesis 1 above is verified. The implementation of internal control enables the management to effectively identify the external environmental risks, opportunities and internal resources through the risk assessment, select those high-quality innovation projects and improve the efficiency of innovation output. To improve the innovation ability of enterprises, it is necessary to attach importance to the construction of internal system. There are many factors affecting the innovation ability of enterprises, but internal control is undoubtedly an important factor [73]. Innovation performance is closely related to the internal resource allocation system of enterprises. It is an important measure to improve the innovation ability of enterprises to attach importance to the micro-level system construction of enterprises, including internal control. The effective internal control prevents various risks in the process of technological innovation through the reasonable risk assessment, control and prevention [15, 16].

#### 5.1.2 Analyses of model 1’s control variables

In the control variables, the coefficient on L.R&D is positive and significant (0.023, *p* < 0.10), indicating that the increase of R&D investment is undoubtedly an important factor to improve innovation performance. Enterprises should formulate their scientific R&D expenditure plans according to the established development strategy. The coefficient on L.ROA is positive and significant (2.103, *p* < 0.01), indicating that the good accounting earnings can provide more financing for innovation investment. The coefficient on DUAL is positive and significant (0.224, *p* < 0.01). When the chairman of the board is also the general manager, the senior executives take their own performance evaluation into consideration and try to avoid the inefficiency of innovative resource input, to improve the performance evaluation of senior executives by stakeholders. The coefficient on LnASSET is positive and significant (0.319, *p* < 0.01), implying that those large-scale enterprises often have strong R&D strength, which is conducive to improving their innovation performance. The estimated coefficients on the other control variables are not statistically significant.

### 5.2 Analyses of model 2’s regression results

#### 5.2.1 Analyses of model 2’s explanatory variable

As shown in column 3 of Table 4, in model 2, the coefficient on the explanatory variable (IC) is positive and significant (0.064, *p* < 0.01), indicating that the improvement of the effectiveness of internal control promotes the performance of CSR activities. Hypothesis 2 above is verified. Enterprises carry out CSR activities through various commercial and social practices, bringing sustainable and just benefits to stakeholders, which promotes the welfare of society, and protects the members of organizations. On basis of the comprehensive monitoring, the effective internal control is adopted to supervise and correct the process of production and operation, so as to ensure that the operational risks are evaded sufficiently. Enterprises strengthen the construction of internal control, and protect the legitimate rights and interests of stakeholders, which to a large extent reflect the basic objectives of social governance. The partial content of internal control, as a specific system of corporate governance, still has a positive effect on the performance of social responsibility [17, 18].

#### 5.2.2 Analyses of model 2’s control variables

In the control variables, the coefficient on L.ROA is positive and significant (1.358, *p* < 0.01), suggesting that the better accounting surplus of enterprises provides more material support for CSR activities, which is conducive to promoting enterprises to carry out CSR activities, which conforms to the resource-based hypothesis. The coefficient on L.LER is positive and significant (0.214, *p* < 0.01), implying that the creditor governance has a positive governance effect on CSR practices. The coefficient on L.GROWTH is positive and significant (0.201, *p* < 0.01), indicating that the better the development prospect of enterprises, the better the performance of social responsibility. The coefficient on SHRCR is positive and significant (0.015, *p* < 0.01). When the degree of equity concentration is high, the interests of major shareholders and the enterprise as a whole tend to be consistent. In order to realize long-term interests, major shareholders tend to encourage enterprises to better carry out CSR activities. Besides, the coefficient on DTL is negative and significant (-0.011, *p* < 0.01), suggesting that for those enterprises with higher operational risks, senior executives should be urged to improve CSR performance on basis of establishing an effective incentive mechanism while realizing entrepreneur profits. And the coefficient on LnASSET is negative and significant (-0.173, *p* < 0.05). While pursuing profits, large-scale enterprises should fully pay attention to the legitimate rights and interests of stakeholders, so as to gain a better consumer trust and social image. The estimated coefficients on the other control variables are not statistically significant.

### 5.3 Analyses of model 3’s regression results

#### 5.3.1 Analyses of the intermediary effect

In model 3, the coefficient on IC is positive and significant (0.040, *p* < 0.10), indicating that the improvement of the effectiveness of internal control will help improve the innovation performance of enterprises. The coefficient on SCPS is positive and significant (0.135, *p* < 0.01), indicating that the strengthening of CSR practices will be a positive driving factor to enhance innovation performance. In addition to economic interests, enterprises should actively respond to the expectations and appeals of different stakeholders, enhance their social reputation and establish a good corporate image. The good social reputation of enterprises will enhance their competitiveness in an invisible way. In order to effectively maintain an enterprise’s competitive advantage, stakeholders supervise and motivate the management to identify the external opportunities and internal resources, formulate the scientific innovation plans, combine the market development with technology development, and then effectively improve their innovation performance. Meanwhile, given that the effect of internal control on social contribution per share is also positive and significant (β_1_, *p* < 0.01), in general, CSR activities take an intermediary effect between internal control and innovation performance. Hypothesis 3 above is verified. In addition to economic interests, enterprises should feedback to stakeholders’ expectations, supervise and motivate the management to make the scientific innovation plans, so as to effectively improve the level of innovation performance. Moreover, the non-parametric percentile Bootstrap method of deviation correction is adopted. After 1000 runs, the confidence interval corrected of β_1_×γ_2_ for 95% confidence is [0.007, 0.028]. Where, β_1_×γ_2_ is the product of the effect of IC on SCPS and that of SCPS on LnPATENT. Moreover, the effect size of the intermediary effect is about 18.78% (i.e. β_1_ ×γ_2_/α_1_ = 0.064×0.135/0.046).

#### 5.3.2 Analyses of model 3’s control variables

In the control variables, the coefficients on L.ROA, DUAL and LnASSET are positive and significant (1.892, *p* < 0.01; 0.218, *p* < 0.01; 0.346, *p* < 0.01). This indicates that these variables have convergent analysis conclusions with the corresponding variables of model 1 in Table 4. The good accounting surplus, appraisal expectations for senior executives and scale advantage are the positive factors to enhance the efficiency of enterprise innovation. The estimated coefficients on the other control variables are not statistically significant.

## 6. Further analysis

According to the composition of listed enterprises in Chinese capital market, state-owned enterprises occupy a dominant position in terms of scale and number [74]. The effect of internal control on innovation input is influenced by the property attributes of enterprises [75]. The operation mode, operation management and supervision of state-owned enterprises and non-state-owned enterprises are not the same, and there are some differences in the efficiency of technological innovation and CSR activities. Therefore, the property attributes are taken as the basis for grouping test. Based on distinguishing the property attributes of different enterprises, this study conducts the further analyses on model 1—model 3.

### 6.1 Further analyses of model 1’s regression results

For model 1, this study distinguishes between the state-owned and non-state-owned samples. LSDV method is used to analyze model 1, and the null hypothesis that “the coefficients of all individual dummy variables are zero” is rejected, indicating the existence of individual fixed effect. The robust Hausman tests for model 1 show that the Sargan-Hansen *χ*^2^ are 231.172 (*p = 0*.*000*), 230.268 (*p = 0*.*000*), respectively, indicating that the fixed effect model should be adopted instead of random effect. Accordingly, the statistical results of the fixed-effect models are shown in columns 2 and 3 of Table 5.

**Table 5:** Statistical results of further analysis for models 1–3.

Variable	Model 1		Model 2		Model 3	
	State-owned	Non-state	State-owned	Non-state	State-owned	Non-state
	Coefficient (S.E.)	Coefficient (S.E.)	Coefficient (S.E.)	Coefficient (S.E.)	Coefficient (S.E.)	Coefficient (S.E.)
Intercept	-6.141* (3.378)	-3.218 (2.094)	3.245 (3.161)	1.450 (2.170)	-6.937** (3.167)	-3.390 (2.087)
IC	0.008 (0.034)	0.068** (0.031)	0.092*** (0.031)	0.048*** (0.018)	-0.003 (0.034)	0.066** (0.031)
SCPS					0.168*** (0.047)	0.086* (0.044)
L.R&D	0.006 (0.022)	0.031** (0.015)	0.014 (0.012)	-0.003 (0.009)	-0.008 (0.035)	0.032** (0.015)
L.ROA	1.525* (0.866)	2.487*** (0.668)	2.155*** (0.616)	0.876* (0.445)	1.019 (0.856)	2.459*** (0.669)
L.LER	-0.020 (0.055)	-0.021 (0.095)	0.214*** (0.073)	0.190*** (0.055)	-0.058 (0.054)	-0.035 (0.097)
L.GROWTH	-0.044 (0.072)	-0.016 (0.071)	0.165** (0.065)	0.225*** (0.045)	-0.087 (0.075)	-0.035 (0.069)
SHRCR	0.017*** (0.006)	-0.004 (0.003)	0.024*** (0.007)	0.008*** (0.002)	0.013*** (0.005)	-0.005 (0.003)
SUPERVISOR	0.006 (0.057)	-0.070 (0.062)	-0.057 (0.056)	-0.005 (0.046)	0.015 (0.060)	-0.070 (0.062)
DUAL	0.035 (0.080)	0.305*** (0.075)	0.010 (0.092)	0.006 (0.042)	0.034 (0.081)	0.300*** (0.075)
L.LNSALARY	0.092 (0.092)	-0.073 (0.087)	0.077 (0.077)	0.083 (0.062)	0.079 (0.092)	-0.079 (0.086)
L.DTL	-0.003 (0.005)	0.004 (0.007)	-0.014*** (0.005)	-0.006* (0.003)	-0.001 (0.005)	0.005 (0.007)
LnASSET	0.292** (0.121)	0.338*** (0.081)	-0.172 (0.118)	-0.105 (0.081)	0.333*** (0.115)	0.350*** (0.081)
L.AUDIT	-0.143 (0.235)	0.102 (0.234)	-0.077 (0.339)	0.029 (0.105)	-0.128 (0.220)	0.086 (0.235)
YEAR/ IND	YES	YES	YES	YES	YES	YES
# of obs.	1524	2066	1521	2065	1521	2065
Within_R^2^	0.132	0.125	0.234	0.110	0.151	0.128
F_Value	7.81***	11.04***	15.65***	9.48***	8.77***	10.75***

*** Significant at 1%

** Significant at 5%

* Significant at 10%.

Robust standard errors in brackets are clustered at the enterprise level.

#### 6.1.1 Further analyses of model 1’s explanatory variable

As shown in columns 2 and 3 of Table 5, in the state-owned sample, the coefficient on the explanatory variable (IC) is not statistically significant (0.008, *p* > 0.10). And in the non-state sample, the coefficient on the explanatory variable (IC) is positive and significant (0.068, *p* < 0.05). These above results suggest that the positive effect of internal control on technological innovation is limited by the property attributes of enterprises, and the improvement of the effectiveness of internal control in state-owned enterprises does not exert a significant effect on the performance of technological innovation. The results show that the enhancement of the effectiveness of internal control is conducive to improving innovation performance, which comes from the supervision and motivation of internal control during the implementation of innovation projects, reducing those inefficient R&D projects, and strengthening the efficiency of innovation activities to improve innovation performance. However, the positive effect of internal control on innovation performance is only prominent in non-state-owned enterprises. And in state-owned enterprises, the implementation of internal control has not yet played an obvious promoting effect on innovation performance. The positive impact of internal control on innovation performance is subject to the property attributes, and the impact of internal control on technological innovation is not significant in the state-owned sample, which may be caused by the unclear ownership and management rights, and the lack of incentive and supervision mechanism [76]. After T test with sample data, it is found that the difference between the mean of internal control index of state-owned enterprises and that of non-state-owned enterprises is 4.927, which is not statistically significant. Therefore, the regulators should urge the state-owned enterprises to play the positive role of internal control in innovation activities, so as to promote the healthier and more stable economic development.

#### 6.1.2 Further analyses of model 1’s control variables

In the non-state sample, the coefficient on L.R&D is positive and significant (0.031, *p* < 0.05). The intensification of R&D investment will be an important factor in improving innovation performance, but this positive effect is only reflected in the non-state sample. The possible reason is that the residual control rights and residual claims of state-owned enterprises are difficult to correspond to the uncertain technological innovation choices, while non-state-owned enterprises can better solve this problem, and the technological innovation efficiency of non-state-owned enterprises is higher than that of state-owned enterprises. In the state-owned and non-state samples, the coefficients on L.ROA are positive and significant (1.525, *p* < 0.10; 2.487, *p* < 0.01). In general, a good accounting surplus is the material guarantee to improve innovation performance. In the state-owned sample, the coefficient on SHRCR is positive and significant (0.017, *p* < 0.01). For state-owned enterprises, it is an effective way to improve innovation performance to enhance the supervision of major shareholders on innovation activities under the circumstance that internal control has not yet exerted a significant promoting effect on innovation performance. In the non-state sample, the coefficient on DUAL is positive and significant (0.305, *p* < 0.01). This result implies that in non-state-owned enterprises, when the chairman is also the general manager, stakeholders have higher expectation on the performance of senior executives, which to some extent promotes senior executives to enhance innovation performance. Besides, in the state-owned and non-state samples, the coefficients on LnASSET are positive and significant (0.292, *p* < 0.05; 0.338, *p* < 0.01). Furthermore, it can be found from the correlation of variables (Table 3) that LnASSET is positively and significantly (0.090, *p* < 0.01) correlated with IC. While paying attention to strengthen the effectiveness of internal control, larger enterprises have effectively improved their innovation performance. Large enterprises often have a strong competitive advantage in the aspects of cost, market share and brand influence.

### 6.2 Further analyses of model 2’s regression results

For model 2, this study distinguishes between the state-owned and non-state-owned samples. LSDV method is used to analyze model 2, and the null hypothesis that “the coefficients of all individual dummy variables are zero” is rejected, indicating the existence of individual fixed effect. The robust Hausman tests for model 2 show that the Sargan-Hansen *χ*^2^ are 156.420 (*p = 0*.*000*), 148.089 (*p = 0*.*000*), respectively, indicating that the fixed effect model should be used instead of random effect. Accordingly, the statistical results of the fixed-effect model are shown in columns 4 and 5 of Table 5.

#### 6.2.1 Further analyses of model 2’s explanatory variable

In the state-owned and non-state samples, the coefficients on the explanatory variable (IC) are positive and significant (0.092, *p* < 0.01; 0.048, *p* < 0.01). The improvement of the effectiveness of internal control promotes enterprises to actively carry out the necessary CSR practices. The effective internal control has a spillover effect, which not only affects the production and operation of enterprises themselves, but also affects all stakeholders. Hypothesis 2 above is verified again. Enterprises should continue to strengthen the internal governance and form an effective system of internal control, which will be an important safeguard for safeguarding the legitimate rights and interests of stakeholders.

#### 6.2.2 Further analyses of model 2’s control variables

In the state-owned and non-state samples, the coefficients on L.ROA are positive and significant (2.155, *p* < 0.01; 0.876, *p* < 0.10). Whether in state-owned or non-state-owned enterprises, the good return on assets is the material premise to engage in CSR activities. The coefficients on L.LER are positive and significant (0.214, *p* < 0.01; 0.190, *p* < 0.01). Creditor governance has a positive effect on CSR practices. And the coefficients on L.GROWTH are positive and significant (0.165, *p* < 0.05; 0.225, *p* < 0.01). The optimistic development prospect of enterprises is often accompanied by the better CSR practices. Also, the coefficients on SHRCR are positive and significant (0.024, *p* < 0.01; 0.008, *p* < 0.01). When the enterprise’s equity is relatively concentrated, the interests of major shareholders and the overall interests of the enterprise are convergent, which improves CSR performance. Besides, the coefficients on L.DTL are negative and significant (-0.014, *p* < 0.01; -0.006, *p* < 0.10). The excessive operational risk will take a negative impact on CSR activities. Therefore, it is a strategic move for the management to control the operation risk at a reasonable level.

### 6.3 Further analyses of model 3’s regression results

For model 3, this study distinguishes between the state-owned and non-state-owned samples. LSDV method is used to analyze model 3, and the null hypothesis that “the coefficients of all individual dummy variables are zero” is rejected, indicating the existence of individual fixed effect. The robust Hausman tests for model 3 show that the Sargan-Hansen *χ*^2^ are 204.646 (*p = 0*.*000*), 212.853 (*p = 0*.*000*), respectively, indicating that the fixed effect model should be adopted instead of random effect. Accordingly, the statistical results of the fixed-effect model are shown in columns 6 and 7 of Table 5.

#### 6.3.1 Further analyses of the intermediary effect

In the state-owned sample, the coefficient on IC is not statistically significant (-0.003, *p* > 0.10). And in the non-state sample, the coefficient on IC is positive and significant (0.066, *p* < 0.05). Once again, the results show that the positive effect of internal control on technological innovation is limited by the property attributes. The internal control of non-state-owned enterprises takes a significant and positive effect on the performance of technological innovation. However, in state-owned enterprises, internal control does not give play to a significant impact on the performance of technological innovation. For state-owned enterprises, the positive effect of internal control on innovation performance should remain the focus of regulators’ work in the future.

In the state-owned sample, the coefficient on SCPS is positive and significant (0.168, *p* < 0.01). In the non-state sample, the coefficient on SCPS is positive and significant (0.086, *p* < 0.10). The enhancement of CSR performance will be an effective driving factor to improve innovation performance. Meanwhile, for model 1, in the state-owned sample, the coefficient on the explanatory variable (IC) is not statistically significant (0.008, *p* > 0.10). And in the non-state sample, the coefficient on IC is positive and significant (0.068, *p* < 0.05). Thus, hypothesis 3 above is verified again. However, the intermediary effect of CSR between internal control and innovation performance is only reflected in the non-state-owned sample. And then, the non-parametric percentile Bootstrap method of deviation correction is adopted. After 1000 runs, in the non-state sample, the confidence interval corrected of β_1_×γ_2_ for 95% confidence is [0.013, 0.048]. Where, β_1_×γ_2_ represents the product of the effect of IC on SCPS and that of SCPS on LnPATENT. Also, the effect size of the intermediary effect is about 6.07% (i.e. β_1_ ×γ_2_/α_1_ = 0.048×0.086/0.068).

#### 6.3.2 Further analyses of model 3’s control variables

For the control variables, in the state-owned sample, the coefficients on SHRCR and LnASSET are positive and significant (0.013, *p* < 0.01; 0.333, *p* < 0.01), implying that the moderate equity concentration and scale advantage are favorable motivations to enhance the efficiency of innovation output in state-owned enterprises. And in the non-state sample, the coefficients on L.R&D, L.ROA, DUAL and LnASSET are positive and significant (0.032, *p* < 0.05; 2.459, *p* < 0.01; 0.300, *p* < 0.01; 0.350, *p* < 0.01), indicating that the abundant R&D resources input, good accounting surplus, performance expectation for senior executives and scale advantage are all positive drivers to strengthen innovation performance in non-state-owned enterprises. This indicates that these above variables have convergent analysis conclusions with the corresponding variables of model 3 in Table 5.

## 7 Robustness tests

The effectiveness of internal control is influenced by the environment and characteristics of the enterprise. And the characteristic variables of enterprises with high effectiveness of internal control and enterprises with low effectiveness are inevitably quite different. If these characteristic variables also affect enterprise innovation performance and CSR performance, the relationship between internal control and innovation performance observed in the previous whole-sample analyses may be interfered with. Therefore, Abadie and Imbens (2006) [77] Matching Estimator is adopted to construct model 4 based on the multiple dimensions of corporate characteristics and governance status, and the Logistic regression is adopted to estimate the propensity score of the effectiveness of internal control (IC). The dummy variable (DIC) of IC is set as the explained variable of Logistic regression. When the actual value of IC is greater than the industry-annual median, the value of DIC is 1; otherwise, it is 0. And then, in addition to the original control variables as covariates, this study refers to the study of Wang et al. (2017) [78], adds the marketization index of the registration place of listed enterprises (Marketization) as a covariate, and screen out the treatment group and control group. Different from the matching of a single indicator, the propensity score matching (PSM) condenses multiple features into a “propensity score value”, thus facilitating the overall matching of multiple features to obtain the same or similar matching samples in terms of the main characteristic variables, so as to reduce the adverse impact of other possible interfering factors on the conclusions. In this study, in accordance with model 4, the Logistic regression is conducted and the propensity scores are calculated. Based on the principle of one-to-one correspondence and near-neighbor matching without replacement, a total of 1536 pairs of paired observations are obtained. And then, the regression analyses of the models are carried out again according to the paired sample.

**Model 4**.

$$\begin{array}{l} {\mathrm{DIC}}_{i,t}=\theta_{0}+\theta_{1}{\mathrm{Marketization}}_{i,t}+\theta_{2}{\mathrm{R\&D}}_{i,t‐1}+\theta_{3}{\mathrm{ROA}}_{i,t‐1}+\theta_{4}{\mathrm{LER}}_{i,t‐1}+\theta_{5}{\mathrm{GROWTH}}_{i,t‐1}+\theta_{6}{\mathrm{SHRCR}}_{i,t}\\ \;+\theta_{7}{\mathrm{SUPERVISOR}}_{i,t}+\theta_{8}{\mathrm{DUAL}}_{i,t}+\theta_{9}{\mathrm{LNSALARY}}_{i,t‐1}+\theta_{10}{\mathrm{DTL}}_{i,t‐1}+\theta_{11}{\mathrm{LnASSET}}_{i,t}+\\ \;\theta_{12}{\mathrm{AUDIT}}_{i,t‐1}+\theta_{13}{\mathrm{STATE}}_{i,t}+\theta_{14}\sum_{t}\mathrm{YEAR}+\theta_{15}\sum_{t}\mathrm{IND}+\varepsilon_{i,t} \end{array}$$ (5)

### 7.1 Robustness tests before properties are distinguished

In view of the differences in the difficulty of technological innovation of enterprises in different industries, it is necessary to consider the influence of industry factors when evaluating innovation performance. Therefore, this study refers to the research design of Li et al. (2018) [51], measures the innovation performance of an enterprise in the current year by dividing the annual number of patent applications by the average of the current year’s patent applications in the industry to which the enterprise belongs, which helps to increase the comparability of innovation output among enterprises in different industries. The specific calculation method is shown in formula (6). Where, M_PATENT represents an enterprise’s annual innovation performance. In the numerator, P represents the number of patent applications in the current period. In the denominator, M_P represents the average number of patent applications of the industry to which the enterprise belongs in the current period, that is, the quotient between the total number of patent applications of a certain industry and the number of individual enterprises in that industry. Furthermore, the calculated results are taken as the explained variable of model 1 and model 3 for the regression analyses again.

M_PATENT=PM_P (6)

Meanwhile, this study takes into account the real social responsibilities of enterprises to stakeholders, which is represented by the cash actually paid to stakeholders under the given income status. Therefore, the research design of Li et al. (2018) [71] is referenced. In this study, the fulfillment of social responsibility (FCSR) is calculated. The specific value of FCSR is the ratio between the cash flow paid for shareholders, creditors, employees, customers, consumers, suppliers, communities, other stakeholders and the average of total shares in the current period. The specific calculation method of FCSR is shown in formula (7). Where, in the numerator of formula (7), M_1_ represents the cash paid to distribute dividends or profits. M_2_ represents the operating cash charges. M_3_ represents the cash paid to pay interest. M_4_ represents the cash paid to and for employees. M_5_ represents the cash paid for goods purchased and services received. And M_6_ represents the cash actually paid for taxes and dues. The denominator S is the average of the total number of shares at the beginning and end of the period. In this way, CSR performance is measured again. The larger the calculated value of FCSR, the higher the performance of CSR activities. Furthermore, FCSR is taken as the explained variable of model 2 and the intermediary variable of model 3 for the regression analyses again.

$$\mathrm{FCSR=}\sum_{i=1}^{6}M_{i}/S$$ (7)

In accordance with the PSM samples, LSDV method is used to analyze model 1—model 3, and the null hypothesis that “the coefficients of all individual dummy variables are zero” is rejected, indicating the existence of individual fixed effect. The robust Hausman tests for model 1- model 3 show that the Sargan-Hansen *χ*^2^ are 107.432 (*p = 0*.*000*), 101.228 (*p = 0*.*000*) and 112.548 (*p = 0*.*000*), respectively, indicating that the fixed effect model should be adopted. Table 6 reports the robustness test results of the whole sample for model 1- model 3.

**Table 6:** Robustness test results before properties are distinguished.

Variable	Model 1	Model 2	Model 3
	Coefficient (S.E.)	Coefficient (S.E.)	Coefficient (S.E.)
Intercept	-8.921*** (2.042)	-0.354 (10.324)	-8.916*** (2.022)
IC	0.025* (0.013)	0.151*** (0.053)	0.022* (0.013)
FCSR			0.015** (0.007)
L.R&D	0.010 (0.010)	0.013 (0.041)	0.010 (0.010)
L.ROA	2.026*** (0.770)	5.619** (2.196)	1.941** (0.767)
L.LER	-0.056 (0.041)	1.335*** (0.347)	-0.077* (0.041)
L.GROWTH	-0.081 (0.074)	1.464*** (0.227)	-0.103 (0.073)
SHRCR	0.006** (0.003)	0.041** (0.020)	0.005* (0.003)
SUPERVISOR	-0.015 (0.051)	-0.103 (0.310)	-0.013 (0.051)
DUAL	0.104*** (0.039)	0.061 (0.211)	0.103*** (0.039)
L.LNSALARY	0.007 (0.060)	-0.238 (0.418)	0.011 (0.060)
L.DTL	-0.010 (0.008)	0.001 (0.016)	-0.010 (0.008)
LnASSET	0.411*** (0.076)	0.261 (0.427)	0.407*** (0.075)
L.AUDIT	-0.131 (0.179)	0.224 (1.004)	-0.134 (0.174)
STATE	0.125 (0.137)	0.127 (0.222)	0.123 (0.136)
YEAR/ IND	YES	YES	YES
# of obs.	3060	3060	3060
Within_R^2^	0.052	0.135	0.056
F_Value	5.46***	15.60***	5.73***

*** Significant at 1%

** Significant at 5%

* Significant at 10%.

Robust standard errors in brackets are clustered at the enterprise level.

As shown in Table 6, in model 1, the coefficient on IC is positive and significant (0.025, *p* < 0.10), showing that the effective internal control is helpful to improve the efficiency of innovation output. Hypothesis 1 above is verified again. In model 2, the coefficient on IC is positive and significant (0.151, *p* < 0.01), implying that the effective internal control is conducive to significantly strengthening the fulfillment of social responsibility. Hypothesis 2 above is verified again.

In the robustness test of model 3, IC and FCSR are de-averaged according to the industry-annual standard, and the interaction term (IC×FCSR) is added to model 3. Furthermore, model 5 (formula 8) with the interaction term is constructed. The coefficient on IC×FCSR is positive and significant (0.003, *p* < 0.10), indicating that there is a synergistic effect between internal control and CSR activities, so it is appropriate to analyze the intermediary effect of CSR activities between internal control and innovation performance. Furthermore, model 3 is re-analyzed by regression. As shown in column 4 of Table 6, the coefficient on IC is positive and significant (0.022, *p* < 0.10). And the coefficient on FCSR is positive and significant (0.015, *p* < 0.01). Moreover, in accordance with the coefficient on IC in model 1, generally, CSR activities have an intermediary effect in the process of internal control to improve innovation performance. Hypothesis 3 above is verified again. After calculation, the confidence interval corrected of β_1_×γ_2_ for 95% confidence is [0.001, 0.028]. Where, β_1_×γ_2_ is the product of the effect of IC on FCSR and that of FCSR on M_PATENT.

**Model 5**.

$$\begin{array}{l} M\_{\mathrm{PATENT}}_{i,t}=\delta_{0}+\delta_{1}{\mathrm{IC}}_{i,t}+\delta_{2}{\mathrm{FCSR}}_{i,t}+\delta_{3}{\mathrm{IC}}_{i,t}\times {\mathrm{FCSR}}_{i,t}+\delta_{4}{\mathrm{R\&D}}_{i,t‐1}+\delta_{5}{\mathrm{ROA}}_{i,t‐1}+\delta_{6}{\mathrm{LER}}_{i,t‐1}+\\ \;\delta_{7}{\mathrm{GROWTH}}_{i,t‐1}+\delta_{8}{\mathrm{SHRCR}}_{i,t}+\delta_{9}{\mathrm{SUPERVISOR}}_{i,t}+\delta_{10}{\mathrm{DUAL}}_{i,t}+\delta_{11}{\mathrm{LNSALARY}}_{i,t‐1}+\\ \;\delta_{12}{\mathrm{DTL}}_{i,t‐1}+\delta_{13}{\mathrm{LnASSET}}_{i,t}+\delta_{14}{\mathrm{AUDIT}}_{i,t‐1}+\delta_{15}{\mathrm{STATE}}_{i,t}+\delta_{16}\sum_{t}\mathrm{YEAR}+\delta_{17}\sum_{t}\mathrm{IND}+\varepsilon_{i,t} \end{array}$$ (8)

For the control variables, in model 1 and model 3, L.ROA, DUAL and LnASSET have convergent conclusions with the corresponding variables in Table 4 above. In model 2, L.ROA, L.LER, L.GROWTH and SHRCR have convergent conclusions with the corresponding variables in Table 4 above. In addition, in model 1 and model 3, the coefficients on SHRCR are positive and significant (0.006, *p* < 0.05; 0.005, *p* < 0.10), implying that the convergence of the interests of major shareholders and the enterprise as a whole will be a favorable factor to improve innovation performance. In model 3, the coefficient on L.LER is negative and significant (-0.077, *p* < 0.10), suggesting that the higher debt pressure will take a negative impact on innovation performance.

### 7.2 Robustness tests after properties are distinguished

In order to ensure the reliability of the analysis results, the measurement methods of core indicators are changed again in the robustness tests for the state-owned and non-state-owned samples. For the explained variable in model 1 and model 3, this study takes into account the provision in the article 22 of the “Patent Law of China” that the inventions and utility models for which the patent rights are granted shall be novel, creative and practical. When an enterprise files an application, the patent can only become the real intellectual property of the enterprise after it is officially authorized. Therefore, based on the research of Feng et al. (2017) [79], and Luong et al. (2017) [80], the number of the annual patent authorizations is used as a measure of innovation performance. Where, the number of patents authorized here refers to the total number of patent applications that are filed by an enterprise and are eventually granted in one year. And this variable is symbolized as “GPATENT”. In the regression analysis, in order to maintain the dimensional consistency, the natural logarithm of 1 plus the variable is taken and denoted as “LnGPATENT”. Moreover, in accordance with the correlation of variables, LnGPATENT is significantly and positively correlated with LnPATENT (0.860, *p* < 0.10), which also ensures the feasibility of introducing LnGPATENT into model 1 and model 3. Meanwhile, for the explained variable in model 2, the studies of Piotroski et al. (2015) [81] and Li et al. (2019) [18] are referenced, the CSR score (HXCSR) published by “Hexun” is adopted as a measure of CSR performance. The “Hexun” is a subsidiary of China securities market research and design center. “Hexun” score is calculated in accordance the annual reports of listed enterprises and CSR reports published by Chinese stock exchanges, and measures CSR performance from five aspects, including the responsibilities to shareholders, employees, supply chain, environment and public welfare. The total score of CSR performance is weighted in the above five aspects, with a maximum of 100 points. The higher the total score, the higher the level of fulfilling social responsibility, and the higher the enthusiasm to take social responsibility. Then, the corresponding variables in the original model 1—model 3 above are replaced again. And then the state-owned and non-state-owned samples are distinguished for the regression analyses.

On the basis of distinguishing the property attributes, in accordance with the PSM samples, LSDV method is used to analyze model 1—model 3, and the null hypothesis that “the coefficients of all individual dummy variables are zero” is rejected, indicating the existence of individual fixed effect. The robust Hausman tests for model 1—model 3 show that the Sargan-Hansen *χ*^2^ are significant and positive statistically (193.713, 80.572, *p = 0*.*000;* 162.191, 74.973, *p = 0*.*000*; 195.990, 81.334, *p = 0*.*000*), respectively, indicating that the fixed effect model should be adopted. Table 7 reports the robustness test results after the property rights are distinguished.

**Table 7:** Robustness test results after property rights are distinguished.

Variable	Model 1		Model 2		Model 3	
	State-owned	Non-state	State-owned	Non-state	State-owned	Non-state
	Coefficient (S.E.)	Coefficient (S.E.)	Coefficient (S.E.)	Coefficient (S.E.)	Coefficient (S.E.)	Coefficient (S.E.)
Intercept	-6.298** (2.585)	-7.640*** (2.312)	-38.723 (53.472)	-1.344 (37.824)	-6.370** (2.582)	-7.637*** (2.319)
IC	0.041 (0.033)	0.058** (0.027)	0.012 (0.900)	1.591** (0.631)	0.041 (0.033)	0.055** (0.028)
HXCSR					-0.002 (0.001)	0.002* (0.001)
L.R&D	0.009 (0.018)	0.038*** (0.011)	0.503 (0.360)	-0.210 (0.244)	0.010 (0.018)	0.038*** (0.010)
L.ROA	0.825(0.754)	0.599 (0.661)	30.992* (16.603)	26.959** (13.165)	0.882 (0.763)	0.549 (0.663)
L.LER	-0.032 (0.049)	-0.085 (0.070)	0.936 (1.001)	1.231 (0.862)	-0.031(0.049)	-0.088 (0.070)
L.GROWTH	-0.011 (0.062)	0.084 (0.059)	3.057 (2.038)	0.344 (1.187)	-0.006 (0.061)	0.083 (0.059)
SHRCR	0.009* (0.005)	-0.0004 (0.003)	-0.003(0.084)	0.132** (0.054)	0.009* (0.005)	-0.001 (0.003)
SUPERVISOR	-0.008 (0.048)	-0.032 (0.069)	0.625 (1.250)	0.068 (1.822)	-0.007 (0.049)	-0.032 (0.069)
DUAL	0.012 (0.070)	0.147** (0.068)	-1.163 (1.953)	1.742 (1.174)	0.010 (0.070)	0.144** (0.069)
L.LNSALARY	0.053 (0.077)	0.067 (0.095)	2.828 (1.835)	-0.433 (1.164)	0.058 (0.076)	0.068 (0.095)
L.DTL	-0.001 (0.004)	0.003 (0.007)	-0.243** (0.119)	-0.212 (0.133)	-0.002 (0.004)	0.004 (0.007)
LnASSET	0.302*** (0.091)	0.435*** (0.082)	0.770 (2.190)	0.956 (1.533)	0.304*** (0.091)	0.433*** (0.082)
L.AUDIT	0.137 (0.306)	-0.147 (0.126)	2.760 (3.373)	-5.214 (7.117)	0.142(0.302)	-0.138 (0.131)
YEAR/ IND	YES	YES	YES	YES	YES	YES
# of obs.	1287	1773	1287	1773	1287	1773
Within_R^2^	0.138	0.124	0.263	0.135	0.140	0.126
F_Value	6.87***	9.78***	15.26***	10.75***	6.70***	9.43***

*** Significant at 1%

** Significant at 5%

* Significant at 10%.

Robust standard errors in brackets are clustered at the enterprise level.

As shown in Table 7, for model 1, in the state-owned sample, the coefficient on IC is not statistically significant (0.041, *p* > 0.10). And in the non-state sample, the coefficient on IC is positive and significant (0.058, *p* < 0.05). Once again, these results show that the positive effect of internal control on technological innovation is restricted by the property attributes of enterprises. This also indicates that state-owned enterprises should strengthen the positive effect of internal control on technological innovation. For model 2, in the non-state sample, the coefficient on IC is positive and significant (1.591, *p* < 0.05). And in the state-owned sample, the coefficient on IC is not statistically significant (0.012, *p* > 0.10). This result is slightly different from the corresponding variable in Table 5 above. The possible reason is that “Hexun” gives a higher evaluation on the role of internal control in strengthening innovation performance in non-state-owned enterprises. For model 3, in the state-owned sample, the coefficients on IC and HXCSR are not statistically significant (0.041, *p* > 0.10; -0.002, *p* > 0.10). And in the non-state sample, the coefficients on IC and HXCSR are positive and significant (0.055, *p* < 0.05; 0.002, *p* < 0.10). Once again, the above results show that the intermediary effect of CSR activities between internal control and innovation performance is only reflected in non-state-owned enterprises. And after calculation, the effect size of the intermediary effect is about 5.49% (i.e. β1×γ2/α1 = 1.591×0.002/0.058).

For the control variables, in model 1 and model 3, L.R&D, SHRCR, DUAL and LnASSET have convergent conclusions with the corresponding variables in Table 5 above. In model 2, L.ROA, SHRCR and L.DTL have convergent conclusions with the corresponding variables in Table 5 above.

## 8 Conclusions and recommendations

### 8.1 Conclusions

From the perspective of the effectiveness of internal control, this study analyzes the impact of internal control on enterprise innovation performance and CSR performance. On this basis, this study analyzes the intermediary effect of CSR activities in the process of internal control influencing innovation performance. The conclusions of the study are as follows. The improvement of the effectiveness of internal control has a significant promoting effect on enterprise innovation performance, which is consistent with the research conclusions of Lu et al. (2011) [73] and Chen et al. (2018) [20]. To improve the innovation ability, enterprises should attach importance to the internal system. And the enhancement of the effectiveness of internal control is undoubtedly an important factor to improve innovation performance. And the improvement of the effectiveness of internal control promotes enterprises to significantly improve CSR performance. Enterprises strengthen the construction of their own internal system and safeguard the legitimate rights and interests of stakeholders, which to a large extent reflect the basic objectives of social governance.

Also, on basis of the literature on the relationship between internal control and innovation performance [19, 20], this study introduces the intermediary variable–CSR performance. The results show that the improvement of CSR performance is a positive driving factor for strengthening innovation performance, and CSR activities take an intermediary effect in the process of internal control to improve innovation performance. The further analyses show that the positive effect of internal control on technological innovation is subject to the property attributes. In state-owned enterprises, internal control does not take the significant and positive effect on innovation performance. And the effective internal control promotes enterprises to significantly improve CSR performance. In non-state-owned enterprises, CSR activities play a partial intermediary effect in the process of internal control to improve innovation performance, but this intermediary effect is not reflected in state-owned enterprises.

In accordance with the existing literature, scholars have expounded the influence of internal control on the risks of technological innovation [15, 16]. Also, scholars have elaborated on the relationship between internal control and CSR activities to a certain extent [17, 18]. Besides, Akisik and Gal (2017) [82] studied the relationship among CSR reports, internal control and financial performance. However, there are few literature on the relationship among internal control, CSR and innovation performance. On basis of the relevant studies of Belloc (2012) [19] and Chen et al. (2018) [20], this study explores deeply the intermediary effect of CSR activities between internal control and innovation performance. This study expounds the mechanism that internal control and CSR influence enterprise innovation performance, and enriches the existing literature on the relationship between internal control and innovation performance. This study has a certain reference value for strengthening the construction of internal control, improving the efficiency of innovation output and strengthening CSR practices under the background of economic transformation and upgrading.

### 8.2 Recommendations

Enterprises should continuously improve the effectiveness of internal control, so as to promote the successful realization of their business objectives and strategic objectives. Within an industry, the more communication and collaboration between state-owned and non-state-owned enterprises should be encouraged to prevent the possible negative impact of rigid control points, budget and performance appraisal system on innovation activities. In the implementation of internal control, the relationship between institutional control and flexibility should be dynamically balanced, the content and proportion of each control element should be refined, and the formalization of institutional construction and management rigidity should be avoided. The effectiveness of internal control should be strengthened, to strengthen the institutional foundation to enhance the innovation vitality of enterprises, and further improve the efficiency of technological innovation.

In the process of improving innovation performance, enterprises should carefully design rules to balance the private and social benefits of innovators. A normative system of CSR information disclosure is constructed from the perspective of the effectiveness of internal control. The regulators encourage auditors and enterprises to add gradually the relevant contents of “CSR assessment” in the audit reports and self-assessment reports of internal control, respectively, so as to enhance the reliability of CSR information disclosure. Moreover, enterprises ensure the effectiveness of internal control, strengthen the practical performance of social responsibility, and improve the protection of stakeholders’ rights and interests. On basis of improving CSR performance, enterprises should establish the good corporate image and deliver the better reputation information to stakeholders, and then obtain and maintain the lasting competitive advantages.

From the long-term perspective of internal control construction, enterprises should make greater efforts in CSR practices. The regulators should promote the joint mechanism of internal control and CSR to protect the legitimate rights and interests of stakeholders. For instance, under the impact of the new coronavirus pneumonia, enterprises should strengthen the institutional responsibility of scientific prevention and control, gradually restore to the normal production and operation, and then promote the economic and social order to return to better normality. Meanwhile, in the process of improving innovation performance through the institutional improvement, the intermediary effect of CSR activities should be promoted. It is necessary to explore the scientific and technological cooperation among those cross-ownership enterprises, identify the opportunities related to CSR strategies, and integrate the R&D behaviors into CSR strategies. Enterprises with different property attributes should work together to establish the R&D centers, make the major technological breakthroughs in R&D activities, improve their innovation performance to reward stakeholders’ expectations and demands, and maintain the close ties with all stakeholders.

## Supporting information

S1 Dataset The data set used in this article for discussion and analysis. (ZIP)
