# Supplementary material for: The effectiveness of internal control and innovation performance: An intermediary effect based on corporate social responsibility
Source: PLoS One. 2020 Jun 11;15(6):e0234506. doi: 10.1371/journal.pone.0234506 (PMC7289431; doi:10.1371/journal.pone.0234506)
Supplement: S1 Dataset — (ZIP) [file pone.0234506.s001.zip › S1/Main regress/Code.doc]

**1. Descriptive statistics**

tabstat LnPATENT SCPS IC RD ROA LER GROWTH SHRCR SUPVERSIOR DUAL LnSALARY DTL LnASSET AUDIT STATE , stat(mean median max min sd count) col(stat)

**2. Correlation coefficient of variables**

pwcorr LnPATENT SCPS IC RD ROA LER GROWTH SHRCR SUPVERSIOR DUAL LnSALARY DTL LnASSET AUDIT STATE, sig star(0.1)

**3. Model Regression Analysis**

**model 1**：

xtreg LnPATENT IC2 L.RD L.ROA L.LER L.GROWTH SHRCR SUPVERSIOR DUAL L.LnSALARY L.DTL LnASSET L.AUDIT STATE dum*, fe r

**model 2**：

xtreg SCPS IC2 L.RD L.ROA L.LER L.GROWTH SHRCR SUPVERSIOR DUAL L.LnSALARY L.DTL LnASSET L.AUDIT STATE dum*, fe r

**model 3**：

xtreg LnPATENT IC2 SCPS L.RD L.ROA L.LER L.GROWTH SHRCR SUPVERSIOR DUAL L.LnSALARY L.DTL LnASSET L.AUDIT STATE dum*, fe r

**4. Further analysis**

**model 1**：

xtreg LnPATENT IC2 L.RD L.ROA L.LER L.GROWTH SHRCR SUPVERSIOR DUAL L.LnSALARY L.DTL LnASSET L.AUDIT dum* if STATE==1, fe r

xtreg LnPATENT IC2 L.RD L.ROA L.LER L.GROWTH SHRCR SUPVERSIOR DUAL L.LnSALARY L.DTL LnASSET L.AUDIT dum* if STATE==0, fe r

**model 2**：

xtreg SCPS IC2 L.RD L.ROA L.LER L.GROWTH SHRCR SUPVERSIOR DUAL L.LnSALARY L.DTL LnASSET L.AUDIT dum* if STATE==1, fe r

xtreg SCPS IC2 L.RD L.ROA L.LER L.GROWTH SHRCR SUPVERSIOR DUAL L.LnSALARY L.DTL LnASSET L.AUDIT dum* if STATE==0, fe r

**model 3**：

xtreg LnPATENT IC2 SCPS L.RD L.ROA L.LER L.GROWTH SHRCR SUPVERSIOR DUAL L.LnSALARY L.DTL LnASSET L.AUDIT dum* if STATE==1, fe r

xtreg LnPATENT IC2 SCPS L.RD L.ROA L.LER L.GROWTH SHRCR SUPVERSIOR DUAL L.LnSALARY L.DTL LnASSET L.AUDIT dum* if STATE==0, fe r
