# Supplementary material for: The effectiveness of internal control and innovation performance: An intermediary effect based on corporate social responsibility
Source: PLoS One. 2020 Jun 11;15(6):e0234506. doi: 10.1371/journal.pone.0234506 (PMC7289431; doi:10.1371/journal.pone.0234506)
Supplement: S1 Dataset — (ZIP) [file pone.0234506.s001.zip › S1/Robust test/Code(Robust test).doc]

**1. PSM**

**psmatch2 t** RD1 ROA1 LER1 GROWTH1 SHRCR SUPVERSIOR DUAL LnSALARY1 DTL1 LnASSET AUDIT1 STATE dum* index, outcome(M_PATENT) n(1) ate ties logit common noreplacement

**2. The whole sample**

Model 1.

xtreg M_PATENT IC3 L.RD L.ROA L.LER L.GROWTH SHRCR SUPVERSIOR DUAL L.LnSALARY L.DTL LnASSET L.AUDIT STATE dum* if _weight==1, fe r

Model 2.

xtreg CSR IC3 L.RD L.ROA L.LER L.GROWTH SHRCR SUPVERSIOR DUAL L.LnSALARY L.DTL LnASSET L.AUDIT STATE dum* if _weight==1, fe r

Model 3.

xtreg M_PATENT IC3 CSR L.RD L.ROA L.LER L.GROWTH SHRCR SUPVERSIOR DUAL L.LnSALARY L.DTL LnASSET L.AUDIT STATE dum* if _weight==1, fe r

**Confidence interval.**

**bootstrap r(ind_eff) r(dir_eff), reps(5000): sgmediation** M_PATENT**, mv(**CSR**) iv(**IC3**) cv(**RD1 ROA1 LER1 GROWTH1 SHRCR SUPVERSIOR DUAL LnSALARY1 DTL1 LnASSET AUDIT1 STATE dum***)**

**estat bootstrap, percentile bc**

**3. Distinguish between state-owned and non-state-owned samples**

Model 1.

(1) state-owned sample

xtreg LnGPATENT IC2 L.RD L.ROA L.LER L.GROWTH SHRCR SUPVERSIOR DUAL L.LnSALARY L.DTL LnASSET L.AUDIT dum* if _weight==1 & STATE==1, fe r

(2) non-state samples

xtreg LnGPATENT IC2 L.RD L.ROA L.LER L.GROWTH SHRCR SUPVERSIOR DUAL L.LnSALARY L.DTL LnASSET L.AUDIT dum* if _weight==1 & STATE==0, fe r

Model 2.

(1) state-owned sample

xtreg HXCSR IC2 L.RD L.ROA L.LER L.GROWTH SHRCR SUPVERSIOR DUAL L.LnSALARY L.DTL LnASSET L.AUDIT dum* if _weight==1 & STATE==1, fe r

(2) non-state sample

xtreg HXCSR IC2 L.RD L.ROA L.LER L.GROWTH SHRCR SUPVERSIOR DUAL L.LnSALARY L.DTL LnASSET L.AUDIT dum* if _weight==1 & STATE==0, fe r

Model 3.

(1) state-owned sample

xtreg LnGPATENT IC2 HXCSR L.RD L.ROA L.LER L.GROWTH SHRCR SUPVERSIOR DUAL L.LnSALARY L.DTL LnASSET L.AUDIT dum* if _weight==1 & STATE==1, fe r

(2) non-state sample

xtreg LnGPATENT IC2 HXCSR L.RD L.ROA L.LER L.GROWTH SHRCR SUPVERSIOR DUAL L.LnSALARY L.DTL LnASSET L.AUDIT dum* if _weight==1 & STATE==0, fe r

**Confidence interval.**

if _weight==1 & STATE==0

**bootstrap r(ind_eff) r(dir_eff), reps(5000): sgmediation** LnGPATENT**, mv(**HXCSR**) iv(**IC2**) cv(**RD1 ROA1 LER1 GROWTH1 SHRCR SUPVERSIOR DUAL LnSALARY1 DTL1 LnASSET AUDIT1 dum***)**

**estat bootstrap, percentile bc**

**4. Interaction effect**

Model 3.

xtreg M_PATENT IC3 CSR MIC_CSR L.RD L.ROA L.LER L.GROWTH SHRCR SUPVERSIOR DUAL L.LnSALARY L.DTL LnASSET L.AUDIT STATE dum* if _weight==1, fe r

bysort IND year: egen MIC3=mean(IC3)

xtset code1 year

gen M_MIC= IC3- MIC3

xtset code1 year

bysort IND year: egen MCSR =mean(CSR)

xtset code1 year

gen M_CSR = CSR - MCSR

xtset code1 year

gen MIC_CSR = M_CSR* M_MIC

xtset code1 year
